# Studies of liver tissue identify functional gene regulatory elements associated to gene expression, type 2 diabetes, and other metabolic diseases

**DOI:** 10.1186/s40246-019-0204-8

**Published:** 2019-04-29

**Authors:** Marco Cavalli, Nicholas Baltzer, Gang Pan, José Ramón Bárcenas Walls, Karolina Smolinska Garbulowska, Chanchal Kumar, Stanko Skrtic, Jan Komorowski, Claes Wadelius

**Affiliations:** 1 Science for Life Laboratory, Department of Immunology, Genetics and Pathology, Uppsala University, Uppsala, Sweden; 2 Department of Cell and Molecular Biology, Computational Biology and Bioinformatics, Uppsala University, Uppsala, Sweden; 3 AstraZeneca, Gothenburg, Sweden; 4 Institute of Computer Science, Polish Academy of Sciences, Warsaw, Poland

**Keywords:** ChIP-seq, T2D, Regulatory SNPs

## Abstract

**Background:**

Genome-wide association studies (GWAS) of diseases and traits have found associations to gene regions but not the functional SNP or the gene mediating the effect. Difference in gene regulatory signals can be detected using chromatin immunoprecipitation and next-gen sequencing (ChIP-seq) of transcription factors or histone modifications by aligning reads to known polymorphisms in individual genomes. The aim was to identify such regulatory elements in the human liver to understand the genetics behind type 2 diabetes and metabolic diseases.

**Methods:**

The genome of liver tissue was sequenced using 10X Genomics technology to call polymorphic positions. Using ChIP-seq for two histone modifications, H3K4me3 and H3K27ac, and the transcription factor CTCF, and our established bioinformatics pipeline, we detected sites with significant difference in signal between the alleles.

**Results:**

We detected 2329 allele-specific SNPs (AS-SNPs) including 25 associated to GWAS SNPs linked to liver biology, e.g., 4 AS-SNPs at two type 2 diabetes loci. Two hundred ninety-two AS-SNPs were associated to liver gene expression in GTEx, and 134 AS-SNPs were located on 166 candidate functional motifs and most of them in EGR1-binding sites.

**Conclusions:**

This study provides a valuable collection of candidate liver regulatory elements for further experimental validation.

**Electronic supplementary material:**

The online version of this article (10.1186/s40246-019-0204-8) contains supplementary material, which is available to authorized users.

## Background

The understanding of the genetics behind the molecular mechanisms involved in many liver and metabolic diseases remains elusive. Genome-wide association studies (GWAS) of diseases and phenotypic traits have been effective in finding association to gene regions but not the functional SNP(s) or the gene(s) mediating the effect [1]. This is likely due to heterogeneity within and between the study groups, for example, due to several functional common variants on a haplotype, common causative variants that differ between populations, or the contribution of rare personal variants [2]. Another major obstacle is the need to study the regulatory mechanism in the proper tissue.

To date, tens of thousands of associations have been reported between variants and diseases [3], but the question arises whether for example the association of a SNP to breast cancer has a biological relevance if it is located in a regulatory element observed in the skeletal muscle. Moreover, gene regulatory mechanisms are often studied in cell lines derived from cancer cells, which have single nucleotide and copy number variants that drive cancer as well as additional genetic aberrations acquired during prolonged culturing. In addition, culturing cells in the lab also changes the expression of many genes and may activate regulatory elements rarely used in physiological conditions or inactive elements with variants driving associations.

The majority of the GWAS top associated variants are located in non-coding regions [4, 5] and often in high linkage disequilibrium (LD) with several other variants, making it difficult to pinpoint the real functional SNP(s). One way to find putative functional variants is to detect regions with allele-specific (AS) binding of transcription factors (TFs) or their surrogate histone modifications, suggesting a different regulatory downstream role based on the individual genotypes. ChIP-seq data for TFs and histone modifications provide snapshots of direct and indirect protein-DNA interactions allowing the identification of heterozygous SNPs with significant allele-specific signals (AS-SNPs).

Here, we present the results of the identification of AS-SNPs using a minimal set of ChIP-seq datasets produced for two histone modifications and one genome architectural protein in human liver tissue, providing a collection of liver-specific candidate regulatory variants for experimental validations.

## Results

**Fig. 1:**
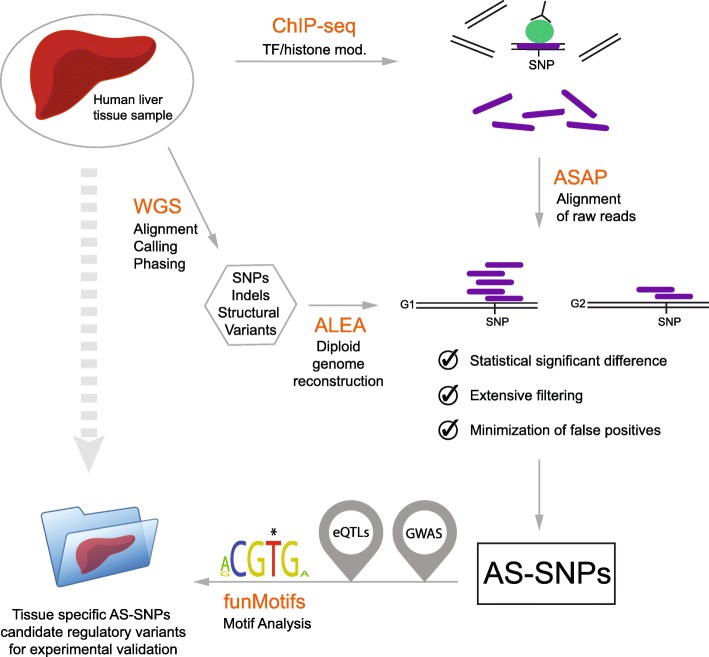
Schematic representation of the study design. Genomic DNA from a human liver sample was submitted to whole genome sequencing (WGS), and the resulting variant calls were used to reconstruct a phased diploid version of the genome and from there two personal genomes (G1 and G2) by replacing the reference bases at heterozygous sites with the alternative alleles using the ALEA software [6]. Chromatin from the liver was used to perform ChIP-seq experiments, and the resulting enriched DNA sequences (purple rods) were sequenced and realigned to the two personal genomes using the ASAP software (http://www.bioinformatics.babraham.ac.uk/projects/ASAP/)

Figure 1 represents the blueprint of this study where we used ChIP-seq data and genomic sequence of a human liver sample to search for AS-SNPs. Initially, the diploid liver genome was reconstructed from the whole genome sequencing SNP calls which yielded a total of 4,588,678 SNPs, 97.8% of which were phased. Two million three hundred thirty-one thousand two hundred two heterozygous SNPs were used to create two personal genomes by replacing the reference bases at heterozygous sites with the alternative alleles. The use of linked-reads in the sequencing step allowed to maintain the correct phasing ensuring the high quality of the two genomes (see the “Methods” section). Using our established bioinformatics pipeline [2], the ChIP-seq reads from CTCF, H3K4me3, and H3K27ac were aligned to the personal genomes to identify heterozygous sites with allele-specific signals and the results were corrected for genome-wide testing. We selected these two established histone modifications which mark active enhancer elements or gene promoters that could act as enhancer elements since most of the reported disease-associated variants in the GWAS catalog are located in non-coding enhancers. CTCF was chosen as an architectural protein involved in the topological organization of the genome. CTCF-binding sites act as “anchor” points for the cohesin complex, and AS-SNPs flagged by a biased alignment of ChIP-seq reads from CTCF could reflect a regulatory activity potentially affecting the structural organization of the genome. We have previously found many allele-specific signals for these histone marks and CTCF in studies of cell lines.

In order to minimize the false positives, we filtered out AS-SNPs located in blacklisted regions from the Encyclopedia of DNA Elements (ENCODE) project or in highly duplicated regions such as those in close proximity to centromeres or telomeres. The liver-specific collection of AS-SNPs obtained in this way consisted of 2329 unique heterozygous SNPs (Additional file 6: Table S2).

### Associations of AS-SNPs to liver-related diseases and gene expression

The AS-SNPs identified by an allele-specific realignment of the ChIP-seq reads define putative regulatory elements that are likely to explain associations to disease in GWAS and to expression in studies of expression quantitative trait loci (eQTLs) (see the section “Genomic features” in the “Methods” section). Therefore, we selected 290 traits/diseases reported in the GWAS catalog and associated to liver activity and metabolism (Additional file 5: Table S1) and the selected AS-SNPs were intersected with the SNPs with the strongest association (GWAS top hit) and with SNPs in high LD (*r*^2^ > 0.8) with GWAS top hits. We identified 25 unique AS-SNPs associated to liver- and metabolic-related traits at 17 different genomic loci (Table 1 and Additional file 7: Table S3) providing new starting points to investigate environmental and gene regulatory signals at these GWAS-defined loci to further clarify the molecular pathways.

**Table 1:** AS-SNPs detected associated to liver-specific GWAS traits

GWAS-associated traits	Number of AS-SNPs	Number of AS loci*
Blood protein levels	5	4
Type 2 diabetes	4	2
Obesity-related traits	3	2
Liver enzyme levels (gamma-glutamyl transferase)	2	1
Fibrinogen levels (smoking status, alcohol consumption, or BMI interaction)	2	1
Cholesterol, total	2	1
Fibrinogen	2	1
Response to hepatitis C treatment	1	1
C-reactive protein levels or total/LDL cholesterol levels (pleiotropy)	1	1
Serum metabolite levels	1	1
Primary biliary cholangitis	1	1
HDL cholesterol	1	1
Total	25	17

*Loci defined as AS-SNPs within 1-Mb regions

For instance, we identified 4 AS-SNPs at two genetic loci associated to type 2 diabetes (T2D) on chromosome 6 and 17. In both cases, the allele-specific signals identified variants that are likely to better explain the associations observed in GWAS.

**Fig. 2:**
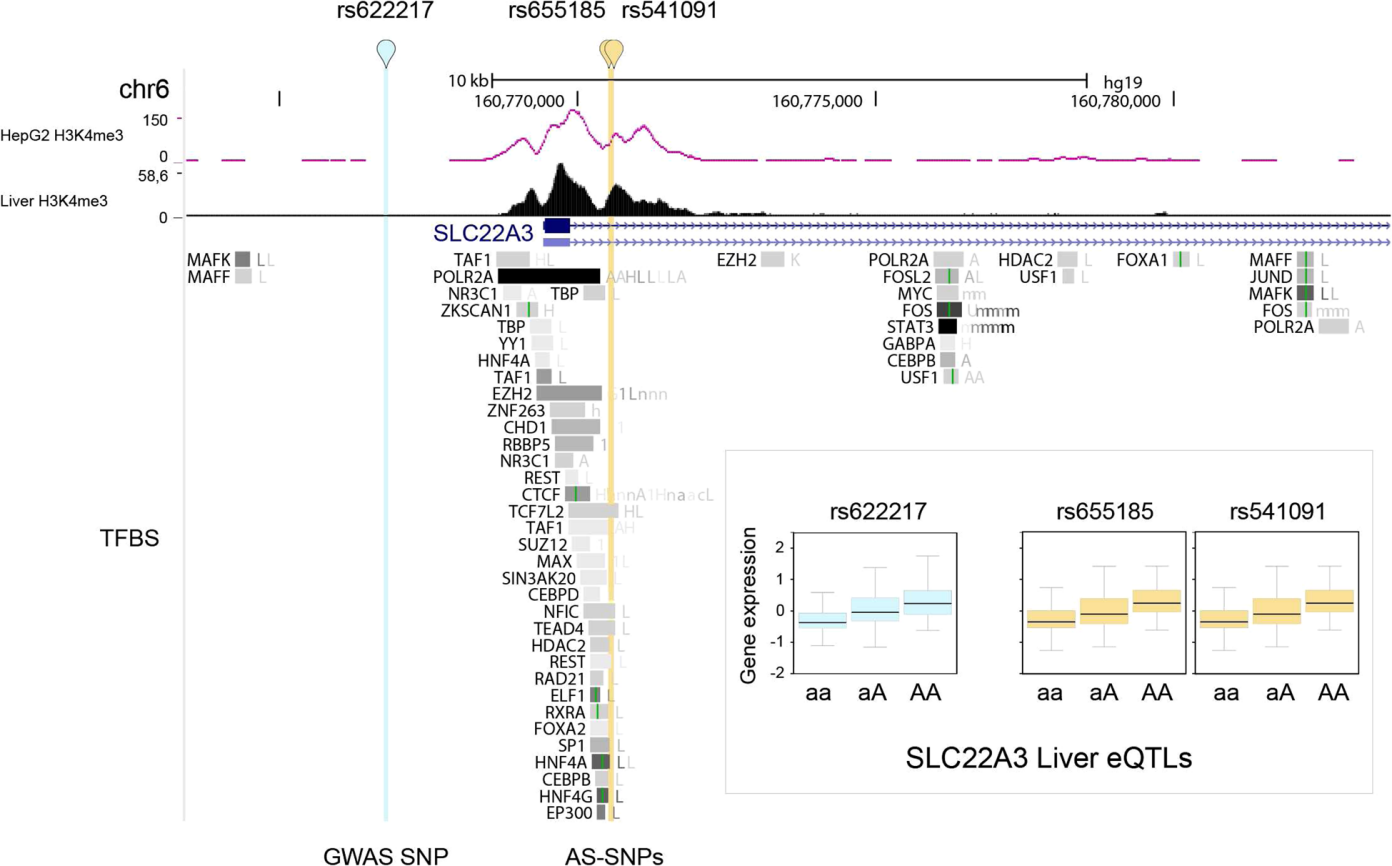
UCSC Genome browser view of the AS-SNPs rs655185 and rs541091 (yellow) which are in high LD with the T2D-associated GWAS SNP rs622217 (cyan). The histone modification tracks represent the peaks called for the ChIP-seq of H3K4me3 performed in the liver (black) and the HepG2 signal from the ENCODE project (pink trace) as a comparison. Transcription factor binding sites (TFBSs) from ChIP-seq data from the ENCODE project for HepG2 and other cell lines. In the bottom insert, eQTL box plots from the GTEx project for the three SNPs with rank-normalized gene expression in liver tissue as a function of the different genotypes

On chromosome 6, two AS-SNPs, rs655185 and rs541091, are in LD with the T2D-associated GWAS SNP rs622217. All three SNPs are reported by Genotype-Tissue Expression (GTEx) as eQTLs for the *SLC22A3* gene in the liver, lung, testis, skin, brain, and esophageal mucosa. However, the analysis of the genetic background revealed that the GWAS SNP rs622217 is not located in a regulatory element due to the lack of transcription factor binding sites (TFBSs) while the two AS-SNPs are located in a regulatory element defined by several TFBSs from ChIP-seq in liver cell lines from the ENCODE project (Fig. 2). The two AS-SNPs are located in the first intron of the *SLC22A3* gene, which encodes for a polyspecific organic cation transporter in the liver, kidney, intestine, and other organs also involved in the uptake of drugs like quinine and metformin, a known first-line medication for T2D. They are also located upstream of the *LPA* and *PLG* genes involved in the regulation of the fibrinolysis and atherosclerosis which have also been linked to T2D and familial hyperlipidemia.

These two AS-SNPs belong to a subset of 9 AS-SNPs (Additional file 8: Table S4) we identified in LD with GWAS SNPs which were also reported as liver eQTLs. This set of AS-SNPs represents candidate regulatory variants supported by LD association to liver-related diseases (GWAS SNPs) and with direct knowledge of the target of gene expression regulation (eQTL SNPs), hence designating ideal candidates for experimental validations.

Another two AS-SNPs associated to T2D were located on chromosome 17, where AS-SNPs rs28528789 and rs62075824 are in LD with the GWAS SNP rs12453394 in a similar fashion to the example reported above for chromosome 6 (Additional file 1: Figure S1). The two AS-SNPs are located in the first intron of the *UBE2Z* gene, which encodes for a ubiquitinating enzyme involved in signaling pathways and apoptosis. The ubiquitin-proteasome system has been suggested to play a role in the process of insulin resistance [7] and diabetes [8], making this locus of particular interest considering the close proximity of other ubiquitin-related genes like *SNF8* and *CALCOCO2*. Another candidate target gene at this locus is *GIP*, which encodes for a potent stimulator of insulin secretion from pancreatic beta cells following food ingestion and nutrient absorption.

Genetic control of gene activity has been analyzed by the GTEx project in different tissues. The significant associations between SNPs and gene expression in the liver were collected from the GTEx project and intersected with the identified AS-SNPs. We found 292 unique AS-SNPs associated to gene expression (Additional file 9: Table S5). The genes with genetically controlled expression and associated to AS-SNPs were highly expressed in hepatocytes (ARCHS4 tissues, GTEx). We observed an enrichment in pathways involved in the regulation of immune response with several AS-SNPs associated to the expression of HLA genes.

### Functional annotations of motifs

In order to obtain TFs active in the liver whose motifs were altered by the functional variants, we overlapped the collection of AS-SNP and TF motifs according to the funMotifs framework (see the “Methods” section). As a result, 595 AS-SNPs were annotated to the TF motifs in liver tissue. We obtained the functional score for each motif using the funMotifs framework. Using the functional score and other parameters introduced for the candidate functional motifs (Umer et al. “funMotifs: Tissue-specific transcription factor motifs”, submitted), we identified 134 variants in 166 functional TF motifs (Additional file 10: Table S6). The majority of TF motifs were located in transcription start site (TSS) regions (Additional file 2: Figure S2). The most recurrent motifs altered by AS-SNPs were observed for the TFs: EGR1, CTCF, KLF5, and ZNF263 (Additional file 3: Figure S3).

The early growth responsive gene-1 (EGR1) is a zinc finger TF that plays an important role in metabolic processes [9] like regulation of cholesterol biosynthesis genes in the liver [10] or insulin resistance in type 2 diabetes [11]. Furthermore, downregulation of EGR1 has been associated to hepatocellular carcinoma (HCC) development [12]. Motifs for EGR3, belonging to the family of EGR1, were also altered by AS-SNPs but to a lower extent.

Krüppel-like factors (KLFs) are TFs that regulate several metabolic pathways, and deregulation of KLFs has been linked to metabolic abnormalities, such as obesity, diabetes, and heart failure [13]. KLF5 has been associated to the onset of fatty liver disease, promoting hepatic lipid accumulation [14]. The motif analysis is aimed at identifying which TF could mediate the effect of the candidate regulatory AS-SNPs potentially altering a downstream gene expression. We identified 13 AS-SNPs altering functional TF motifs which are also reported as liver eQTLs in the GTEx catalog (Additional file 10: Table S6). One example is rs4886705 that is a reported eQTL for the *MAN2C1* gene (Additional file 4: Figure S4).

*MAN2C1* encodes for an enzyme involved in the catabolism of cytosolic-free oligosaccharides, which are released from the degraded proteins. Overexpression of *MAN2C1* has been linked with high mannose levels in the cytosol that could interfere with glucose metabolism [15]. The AS-SNP rs4886705 (A/G) alters a motif for HINFP, a zinc finger TF that interacts with the histone deacetylase complex and plays a role in transcription repression. It may potentially affect the repression of *MAN2C1* leading to metabolic imbalance.

## Discussion

We identified 2329 heterozygous SNPs in a human liver sample that marked putative regulatory elements in the genome based on allele specificity measured through ChIP-seq experiments for histone modifications and TFs. Previous studies have indicated the extreme cell and tissue specificity of these regulators that can be active in one tissue and inactive in others, showing that experimental validations should be performed in the pertinent tissue [16]. At the same time, it is worth to note that gene regulatory mechanisms are often studied in cell lines derived from cancer cells [4]. These cell lines have single nucleotide and copy number variants that drive cancer and additional aberrations acquired during prolonged culturing, and all these variants could bias the interpretation of the molecular mechanisms [17]. Here, we report a collection of variants that flag candidate regulatory elements for liver- and metabolic-related diseases identified in the pertinent tissue context, a healthy human liver sample. Based on the Hardy-Weinberg equilibrium, 33% of common polymorphic sites are heterozygous in one person so in fact we interrogate a reasonable fraction of functional gene regulatory elements that are present in the liver.

The intersection of the liver-specific AS-SNP collection with GWAS and eQTL SNPs was aimed at adding a biological relevance layer. As observed before, the SNPs reported in GWAS and expression studies were directly supported by allele-specific signals in less than 10% of the cases on average. The vast majority of the identified AS-SNPs were in LD with reported GWAS or eQTL SNPs and likely to be the regulatory variants driving the associations. We identified 25 and 292 unique AS-SNPs associated to diseases of the liver and metabolism and gene expression respectively, providing new insights into the molecular regulatory mechanisms. AS-SNPs identified in the human liver flagged 17 genomic loci for several different liver-related traits and diseases.

An example is two loci on chromosome 6 and 17 associated to T2D where AS-SNPs can help explain the association observed in GWAS. The integration with GTEx expression data suggested that the regulatory mechanisms at these loci could link T2D to less familiar pathways such as cationic transporters and drug uptake on chromosome 6 and ubiquitination on chromosome 17.

We also intersected the collection of AS-SNPs with significant variant-gene associations from the GTEx project. We observed a significant number of candidate AS-SNPs (~ 17%) associated to the expression of HLA genes that are expressed not only in immune cells but also in most other tissues and cells. Several experimental and clinical studies have shown how inflammation and tumor progression are working synergistically [18]. An alteration of HLA gene expression can result in losing the ability to present antigens which have been reported to facilitate the metastatic process in cancer cells [19]. Moreover, HLA genes are overexpressed in hepatocytes of the liver with chronic damage or inflammation [20, 21].

The motif analysis was aimed at identifying possible mediators of the regulatory functional activity at the selected AS-SNPs. The rationale is that in altering the sequence of TF binding motifs the AS-SNPs could affect the expression of a target gene. We used functional motif definitions that overlay TFBS with several experimental datasets (e.g., ChIP-seq data, DHSs, CAGE peaks, and TF expression data) going beyond a simple coordinate overlapping with reported TFBS defined from PWMs. We intersected our liver-specific collection of AS-SNPs with functional motifs and found 134 AS-SNPs altering 166 defined functional motifs mostly for TFs expressed in the liver and associated to liver metabolic pathways and development of hepatocellular carcinoma (HCC), such as TFs belonging to the EGR and KLF families. The functional motifs were defined in HepG2 cells, an HCC-derived cell line. This could have led to a definition of more liver cancer-specific motifs and represents a limitation of the method. However, the definition of the functional motifs in the pertinent liver tissue context compensates for the lack of available genomic datasets for human liver tissue. The majority of altered functional motifs were located in the TSS, in agreement with the nature of the allele-specific signal defining the altering AS-SNPs, which in most cases was H3K4me3, a histone modification marking promoters. Finally, we identified 13 AS-SNPs that altered functional motifs and were also associated to gene expression in the liver in the GTEx project. This subset of AS-SNPs represents an excellent starting point for experimental validation of a possible molecular mechanism of gene regulation offering an educated guess on the target (eQTL) and the mediator (funMotifs) of the regulatory process.

## Conclusions

In conclusion, we presented a systematic strategy to find functional gene regulatory variants, the TFs that bind differentially between alleles, and possible target genes in human liver tissue. The collection of AS-SNPs presented here offers a set of candidate regulatory variants supported by several layers of evidence to prioritize experimental validations aimed at improving the knowledge of the molecular mechanisms of many metabolic and liver diseases.

## Methods

### Liver WGS and creation of diploid genome reconstruction

Human liver tissue was obtained from Prof. Per Artursson, Uppsala University. The whole genome of the liver tissue was sequenced using the 10X Genomics technology that relies on linked-reads to provide long-range information usually missing from standard approaches, such as phasing and resolution of haplotypes and structural variants. Genomic DNA was extracted from the liver sample and sequenced to a 36x mean depth coverage. The Chromium™ Software Suite was used to analyze (Long Ranger) and visualize (Loupe) the linked-read sequencing data. We use the diploid genome reconstruction module from the ALEA toolbox [6] that takes a list of phased variants and a reference genome as the inputs. We utilized the variants called by Long Ranger with a “PASS” quality and the Genome Reference Consortium Human Genome Build 37 (GRCh37) as the backbone reference to build two in silico personal genomes for this specific liver sample. ChIP-seq reads aligning to the reference and alternative genomes are referred to as G1 and G2 in Fig. 1 and Additional file 7: Table S3, Additional file 8: Table S4, Additional file 9: Table S5.

### ChIP-seq data

Aliquots of the tissue were grinded to a powder with liquid nitrogen, and ~ 40 mg or ~ 200 mg was utilized to prepare chromatin for histone modifications or TF ChIP using the Diagenode iDeal ChIP-seq kits for histones or TFs, respectively. We performed ChIP for two histone modifications: H3K27ac and H3K4me3, and a genome architectural protein: CTCF. Libraries were prepared from the enriched chromatin with NEBNext Ultra II DNA Library Prep Kit for Illumina (E7645S, NEB) following instructions from the manufacturer and sequenced on HiSeq 2500 system with 100-bp pair end sequencing (Macrogen). The read quality was assessed using Phred64/33 scores with a quality cutoff requirement of 20.

### AS-SNP definition

The AS-SNP discovery was adapted from our established modular pipeline [2] (available on http://bioinf.icm.uu.se/repositories.php, AS-SNP pipeline). In summary, (I) it realigns the ChIP-seq reads to two personal genomes derived from the reconstructed diploid genome, (II) it identifies heterozygous SNPs where the aligned read count differs statistically between the alleles, and (III) it filters out SNPs in blacklisted and duplicated genomic regions. All settings and controls are handled via a single configuration across modules.

### Motif analysis

The potentially functional TF motifs were identified using the funMotifs framework that collected TF motif annotations across the non-coding regions of the human genome in a tissue-specific manner (Umer et al. “funMotifs: Tissue-specific transcription factor motifs”, submitted http://bioinf.icm.uu.se:3838/funmotifs/). The AS-SNPs were overlaid onto the predefined TF motifs for each set of annotations received from a various data types: TF ChIP-seq data, DHSs, CAGE peaks, TF expression data, chromatin state, and information about replication domains. Annotations for the liver were obtained mainly from the HepG2 cell line data. For each of the TF motifs, a functionality score based on the weighted annotations was estimated. The TF motif was indicated as a candidate functional if the DNaseI signal was present on the motif, the TF was expressed, and the motif matching score changed at least 0.3. Furthermore, we required the TF binding event or the significant high functional score for the motif (no less than 2.55).

### Genomic features

AS-SNP collections were intersected and filtered using several publicly available databases:

- GWAS SNPs associated to selected liver- and metabolic-related traits from the NHGRI GWAS catalog [3]. A total of 5051 unique GWAS SNPs were retrieved in addition to 56,958 SNPs in high LD (*r*^2^ > 0.8) with them. A comprehensive list of the selected traits is reported in Additional file 5: Table S1
- Collections of eQTL SNPs from the GTEx project. eGenes and significant variant-gene associations based on permutations in the liver (GTEx Analysis v7 eQTL) were obtained for a total of 290,178 significant associations
- 1000 Genomes SNP collection (1000 Genomes project, phase3-shapeit2-mvncall-integrated-v5a.20130502)
- List of signal artifact blacklisted ENCODE regions [22], centromeric and telomeric regions
- RegulomeDB [23]
- ChromHMM [24] segmentations for the liver tissue from the Roadmap Epigenomics Projects (E066_25_imputed12marks_mnemonics.bed)

## Additional files

Additional file 1: **Figure S1.** UCSC Genome browser view of the AS-SNPs rs28528789 and rs62075824 (yellow) which are in high LD with the T2D-associated GWAS SNP rs12453394 (cyan). TFBS from ChIP-seq data from the ENCODE project for HepG2 and other cell lines. (TIF 1913 kb)

Additional file 2: **Figure S2.** Distribution of TFBS altered by AS-SNPs in different ChromHMM chromatin states. (TIF 260 kb)

Additional file 3: **Figure S3.** Transcription factors whose binding motifs were most frequently altered by AS-SNPs. (TIF 202 kb)

Additional file 4: **Figure S4.** UCSC Genome browser view of the AS-SNPs rs4886705 (yellow) which is a reported eQTL in the liver for the MAN2C1 gene (GTEx eQTL box plot). The histone modification tracks represent the peaks called for the ChIP-seq of H3K4me3 performed in the liver (black) and the HepG2 signal from the ENCODE project (pink trace) as a comparison. TFBS from ChIP-seq data from the ENCODE project for HepG2 and other cell lines. In the bottom panel, the motif for the HINFP transcription factor that is altered by rs4886705. (TIF 2872 kb)

Additional file 5: **Table S1.** List of liver-related traits selected from the GWAS catalog. (XLSX 29 kb)

Additional file 6: **Table S2.** Full collection of liver-specific AS-SNPs. (XLSX 187 kb)

Additional file 7: **Table S3.** Collection of AS-SNPs associated to GWAS SNPs. (XLSX 16 kb)

Additional file 8: **Table S4.** Collection of AS-SNPs associated to GWAS SNPs and reported liver eQTL in GTEx. (XLSX 12 kb)

Additional file 9: **Table S5.** Collection of AS-SNPs reported as liver eQTL in GTEx. (XLSX 50 kb)

Additional file 10: **Table S6.** Results of the funMotifs analysis. (XLSX 16 kb)
